# Input attributes optimization using the feasibility of genetic nature inspired algorithm: Application of river flow forecasting

**DOI:** 10.1038/s41598-020-61355-x

**Published:** 2020-03-13

**Authors:** Haitham Abdulmohsin Afan, Mohammed Falah Allawi, Amr El-Shafie, Zaher Mundher Yaseen, Ali Najah Ahmed, Marlinda Abdul Malek, Suhana Binti Koting, Sinan Q. Salih, Wan Hanna Melini Wan Mohtar, Sai Hin Lai, Ahmed Sefelnasr, Mohsen Sherif, Ahmed El-Shafie

**Affiliations:** 1 Institute of Research and Development, Duy Tan University, Da Nang, 550000 Vietnam; 2 State Commission for Dams and Reservoirs, Ministry of Water Resources, Baghdad, Iraq; 3 Civil Engineering Department El-Gazeera High Institute for Engineering Al Moqattam, Cairo, Egypt; 4 Sustainable Developments in Civil Engineering Research Group, Faculty of Civil Engineering, Ton Duc Thang University, Ho Chi Minh City, Vietnam; 5 Institute of Energy Infrastructure (IEI), Civil Engineering department, Universiti Tenaga Nasional, Kuala, Lumpur Malaysia; 6 Department of Civil Engineering, Faculty of Engineering, University Malaya, Kuala, Lumpur Malaysia; 7 Civil and Structural Engineering Department, Faculty of Engineering and Built Environment, University Kebangsaan Malaysia, Kuala, Lumpur Malaysia; 8 National Water Center, United Arab Emirate University, P.O. Box, 15551 Al Ain, UAE

**Keywords:** Environmental impact, Hydrology

## Abstract

In nature, streamflow pattern is characterized with high non-linearity and non-stationarity. Developing an accurate forecasting model for a streamflow is highly essential for several applications in the field of water resources engineering. One of the main contributors for the modeling reliability is the optimization of the input variables to achieve an accurate forecasting model. The main step of modeling is the selection of the proper input combinations. Hence, developing an algorithm that can determine the optimal input combinations is crucial. This study introduces the Genetic algorithm (GA) for better input combination selection. Radial basis function neural network (RBFNN) is used for monthly streamflow time series forecasting due to its simplicity and effectiveness of integration with the selection algorithm. In this paper, the RBFNN was integrated with the Genetic algorithm (GA) for streamflow forecasting. The RBFNN-GA was applied to forecast streamflow at the High Aswan Dam on the Nile River. The results showed that the proposed model provided high accuracy. The GA algorithm can successfully determine effective input parameters in streamflow time series forecasting.

## Introduction

## Background

The inflow parameter is a significant component of the hydrological process in water resources. Accurate forecasting of river flows for long-term and short-term forecasts are crucial to solving different water engineering problems (e.g., designing agricultural land and flood protection works for urban areas)^1^. Accurate and reliable flow forecasting is a vital reference for making decisions in reservoir system control. Hence, streamflow forecasting modeling has attracted attention and great advances in this field have been developed in recent decades^2^.

Conventional models (linear models) cannot capture the non-linearity and non-stationary of hydrological applications. The autoregressive moving average (ARMA) model, autoregressive model, and autoregressive integrated moving average (ARIMA) model are linear models that have been applied in hydrological time series forecasting^3–5^. The need for determining models capable of addressing the nonlinearity and non-stationary that are characteristics of natural reservoir inflow data has led researchers to propose advanced methods^6,7^. Recently, artificial intelligence methods showed relatively good forecasting accuracy. However, they had trouble detecting the highly stochastic pattern of the data.

The most popular example of artificial intelligence methods is the artificial neural network (ANN). Wu *et al*.^8^ established the Feed Forward Neural Network (FFNN) model for streamflow simulation. The finding evidenced the potential of the FFNN model for streamflow modeling. Two algorithms including multilayer perceptron (MLP) and radial basis function neural network (RBFNN) developed for river flow prediction^9^. The authors reported that the MLP model outperformed the RBNN model. Danandeh Mehr *et al*.^10^ investigated the ability of successive station forecasting models using ANN in a rain gauge-poor watershed as a practical alternative for streamflow prediction. The literature showed that ANNs have disadvantages and limitations including slow learning speed, local minima, a human intervention such as the learning rate and the over-fitting problem. In addition, the modellers of ANN models experienced major difficulties in selection of the proper input pattern for the model to achieve a high level of forecasting accuracy and use the trial and error method to handle the input selection step.

Another example of artificial intelligence method is the Support Vector Machine (SVM) model. The SVM is a statistical learning algorithm used for regression and classifications^11^. Many studies investigated the ability of SVM in streamflow forecasting, such as^12–17^ explored the efficiency of SVM for rainfall-runoff modeling. Daily precipitation, streamflow, and evaporation were used as the input variables for the modeling. Sivapragasam & Liong^18^, investigated the ability of the SVM method to predict streamflow Asefa *et al*.^12^ used SVM to predict seasonal and hourly multi-scale streamflow.

Fuzzy set theory has been popularized as a method for streamflow forecasting in several research studies such as^19–23^. The main advantage of using a fuzzy system is considering the uncertainties in the modeling variables^24,25^. Different fuzzy-based models such as gradient least squares, batch least squares and adaptive neuro-fuzzy system (ANFIS) have been used in modeling engineering systems. The adaptive neuro-fuzzy inference system (ANFIS) model was used by Ahmed El-Shafie *et al*.^20^ to forecast monthly basic inflow. In these models, a special pre-processing for the input pattern is integrated with the basic predictor model. In addition, an input pattern selection procedure based on traditional linear methods such as the correlation between the desired model output and the possible input variables is performed. In these linear methods, this procedure is basically an initial separate step prior to developing the predictive model. Hence, this approach can select a proper input pattern, but another input option may provide better results. Therefore, prior selection of the input pattern before developing the model using the correlation procedure is preferred over trial and error but is still lacking for the optimal selection for the input.

## Problem Statement

The most essential step in developing a forecasting model is the selection of optimal input combinations, as proper input combinations lead to better forecasting accuracy. This step is considered challenging for modellers. In this context, several methods that mainly rely on the correlation between the input and output patterns have been applied. Generally, using the correlation concepts to determine the best input combinations for modeling is not accurate because the correlation between two different variables is based on how strong the linear relation between the variables is, without considering the nonlinear relation. Hence, it is critical to feed the streamflow forecasting modeling the optimal input combinations using methods that can consider the nonlinearity relationship between the variables. In this context, there is a need to develop a special algorithm that can detect and select the optimal input pattern to develop a forecasting model for streamflow at a point along a river. Such an algorithm could search for the optimal input pattern to achieve high forecasting accuracy.

Radial Basis Neural Network (RBNN) is a common method that applied as a predictor in several fields of mechanical, structural, physical, chemical and environmental using a simple and effective relation compare to the artificial intelligent-based neural network^7,24,26–31^. In some time series problem the use of single predictor such as RBNN it might not promise to provide accurate results. Therefore, it is essential to combine it with an optimizer to enhance the performance of prediction. Genetic Algorithm (GA) is one of the robust optimization approach^32^. The algorithm is developed to solve complex engineering problems based on the nature-inspired manners. Recently, GA is advanced to be implemented for diverse engineering applications and real word problems^33–35^.

This study is an integration of the radial basis neural network and genetic algorithm for better model input selection. The search algorithm (i.e., GA) is employed to determine the proper input variables for the predictive model (i.e., RBNN) to achieve a significant level of prediction accuracy.

## Research Innovation

The integration between the Artificial Intelligent (AI) models (including the RBF) and the Genetic Algorithm (GA) has been developed in several prediction/forecasting engineering applications. In these existing models, the GA “as optimizer” has been integrated with the AI model “as predictor” in order to optimize the internal parameters of the AI model’s architecture. The main purpose for the GA in such models is to assure that the convergence process “Mean Square Error (MSE) between the model output and the desired value” is appropriately improved during the training stage and MSE value is decreased through the iteration sequence. In addition, the GA, in a few cases, could fasten the convergence process which is suitable for real-time prediction/forecasting application.

In the current study, on the top of the above benefit of integrating the GA with AI models, the GA has been employed to solve one of the vital challenges in developing AI models which is the model’s input selection. In this study, the GA “as optimizer” has been employed to select the optimal input pattern in order to achieve higher accuracy for the desired model output. In fact, there are unlimited number of combinations for the model’ input could be used to predict the desired output, however, only one of these combinations is the best to achieve the optimal output accuracy. Therefore, in this research, the GA has been utilized to search for the optimal combination for the model’s input that would lead the highest prediction accuracy over the other combinations. In addition, applying this modelling concept for river streamflow forecasting is considered as a new application for such modelling structure. The challenge of such application is that the river streamflow is very highly stochastic and nonlinear pattern. Furthermore, the fact that the used data in this study is monthly river streamflow records for 130 years that experienced enormous patterns of streamflow consequences that ranged between being high “flood”, medium and low “drought” flow added more difficulty to achieve accurate prediction model. In this context, there is a need to develop a new structure for the prediction model that based on optimizing the model’ input pattern to achieve optimal accuracy.

## Research Objectives

The current study focuses on the potential of utilizing Genetic Algorithm (GA) as a selection algorithm to determine the optimal input pattern for a streamflow forecasting model. GA was integrated with the radial basis neural network model and applied for streamflow forecasting at Aswan High Dam (AHD), Egypt. A comprehensive analysis of the forecasting accuracy utilizing the GA-RBNN was conducted. In addition, a discussion of the performance of the proposed GA-RBNN for low, medium and high streamflow patterns is reported.

## Case Study

**Figure 1:**
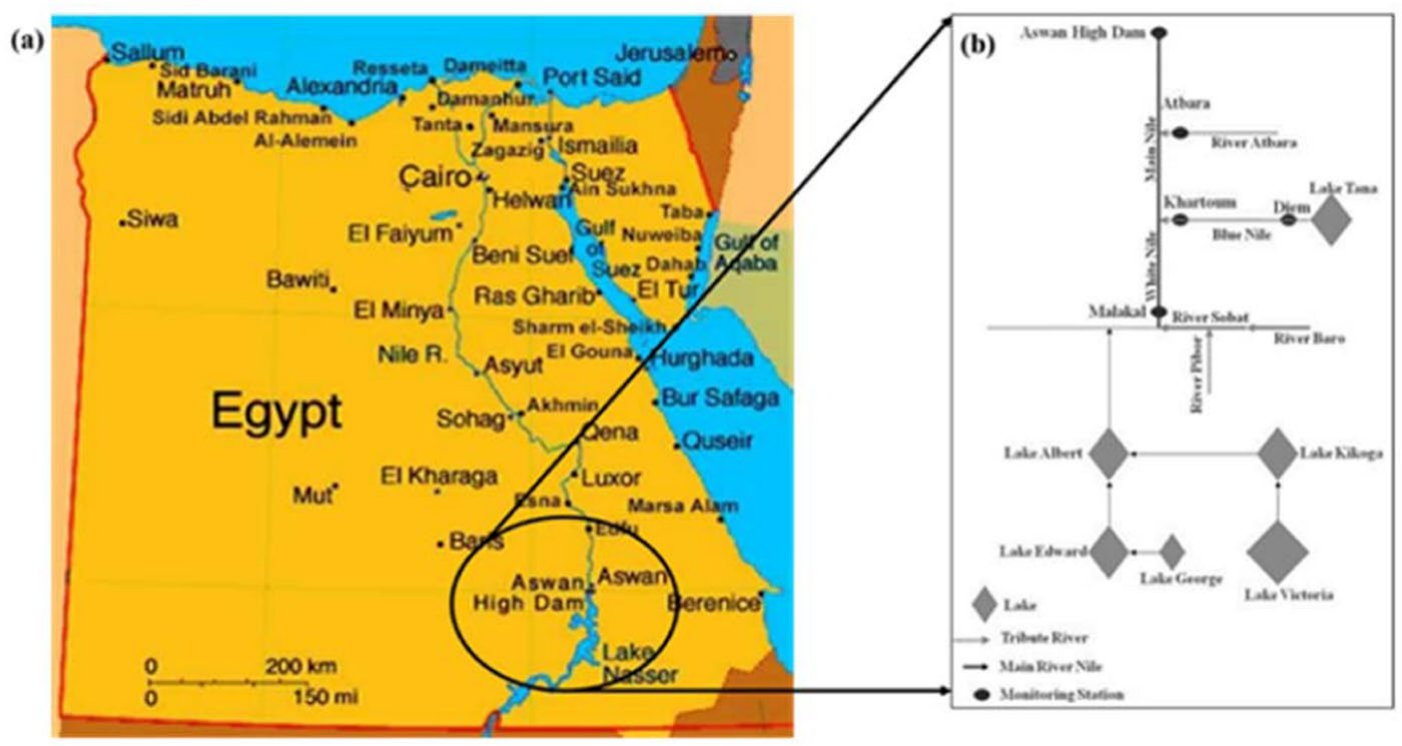
The location of the case study (Aswan High Dam).

One of the longest rivers in the world is River Nile. This River covered a length of about 6850 km, flowing from the south to the north, and lie over 35° of latitude. It has an area of 2.9 Million km^2^ and a catchment basin covering almost 10% of the African continent^36^. Among the major river basins, River Nile is the most complex owing to its massive size (extended through eleven countries) and climatic and topographic variations. The Blue Nile and the White Nile are the two major rivers that makeup River Nile. The Blue Nile (about 1450 km long) has its origin at Lake Tana in Ethiopia; it has a highly stochastic monthly flow. For the White Nile, it originates at Lake Victoria (3700 km long) and characterized by a relatively stable monthly and annual flow. About 80% of the total yearly streamflow of the Blue Nile throughout the rainy season (from July to August) in the Ethiopian highlands is received at the AHD. Over the years, Ethiopia has put up several dams and other flow support structures to dampen the River flow. Egypt and Sudan have since 1902 developed several dams along the River lane. From 1902, the natural inflow has been directly calculated from the general relationship between the stage and discharge in Aswan. This is done by correcting the inflow due to the effect of upstream reservoir losses, deductions in Sudan, and the regulatory effect by the Senna Reservoir. The Aswan High Dam (AHD) is one of the major dams on the Nile River which was constructed to provide Egypt long-term protection against flood and drought. In Egypt, Lake Nasser is the name given to the reservoir formed by the AHD (Fig. 1). The volume of this reservoir is about 160 Billion Cubic Meters (BCM) of stored water. The area studied in this work is the High Aswan Dam located in southern Egypt, along the Nile River. Two mountainous plateaus characterized the River Nile basins; these plateaus peaked at several kilometers above mean sea level and about a thousand kilometers away from AHD (Fig. 1b). The Equatorial or Lake Plateau is situated between the 2 arms of the Great Rift in the southern part of the Nile basin. This lake is about 1,000 to 2,000 m long with peaks of 5,100 and 4,300 m. The eastern part of the basin is formed by the Ethiopian or Abyssinian Plateau, which peaks at about 3,500 m. The basin slopes gradually at the north of the Lake plateau into the Sudan plains. At this plain, the Nile has a lower altitude of fewer than 500 m in its north-wise direction and reaches the AHD^36^. Because the whole natural streamflow to AHD is the result of the rainfall on two mountain plateaus a thousand kilometres away from the AHD, we do not have access to the rainfall events. Therefore, the current study considered a 130-year time series of streamflow data to develop the forecasting model.

**Figure 2:**
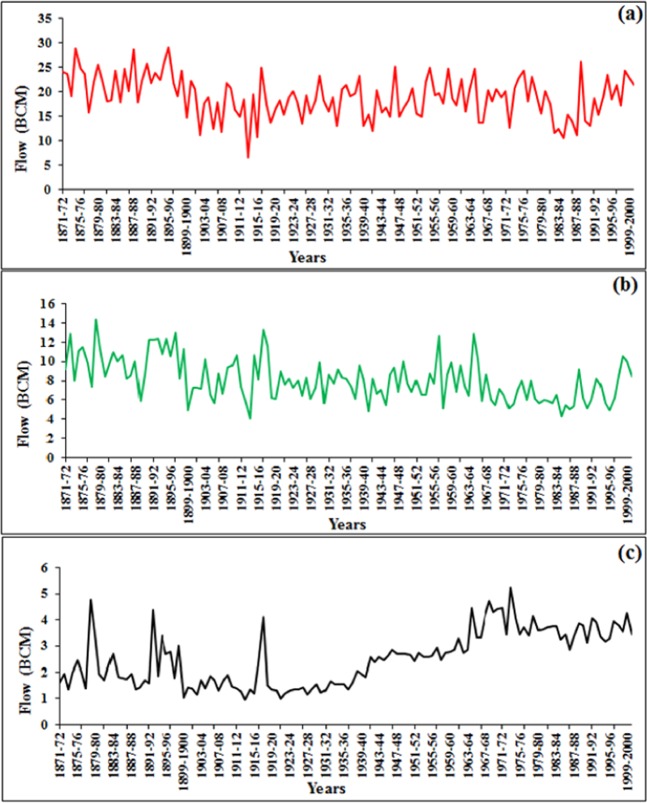
The Natural stream-flow at AHD for months of (**a**) August, (**b**) November and (**c**) April for years between 1870 and 2000.

The Nile River streamflow data in the AHD published by the Egyptian Ministry of water resources and Irrigation from 1871 to 2000 were used in this study. During the 130 years, the natural flow was stochastic. The range of the flow was different from month to month, as shown in Fig. 2. The maximum annual period for inflow recorded at the AHD is from August until October; for example, the streamflow for August was 6.5 to 29 BCM, as shown in Fig. 2a. There were medium flow values in November, December, and January; Fig. 2b shows the natural streamflow for November of 4.12 to 14.4 BCM. The minimum range of the inflow at AHD occurred from February to July; as an example, Fig. 2c shows that the April values were 1 to 5 BCM. The variation of streamflow is a natural phenomenon that can be affected by many factors such as climate conditions, land use, topography, and soil type. All these factors have its own direct and indirect effects on the streamflow such as land use which has direct effects on the surface runoff and indirect such as the evaporation^37^. The nonlinearity of these factors over the years cause a change in the streamflow pattern from year to another. This change can be seen clearly seen over the time series where the streamflow is gradually increasing from 1923 to 1963.

## Genetic Input Selection Model

**Figure 3:**
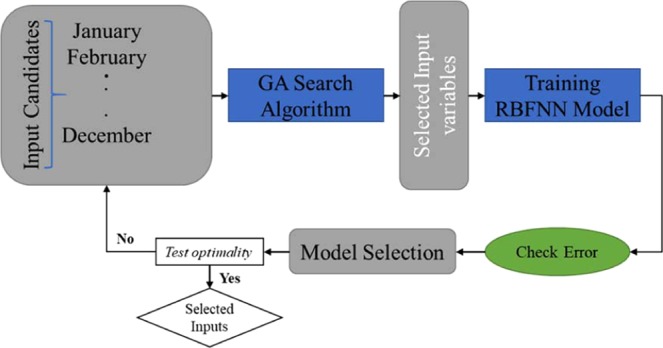
The conceptual input variables selection by using GA.

**Figure 4:**
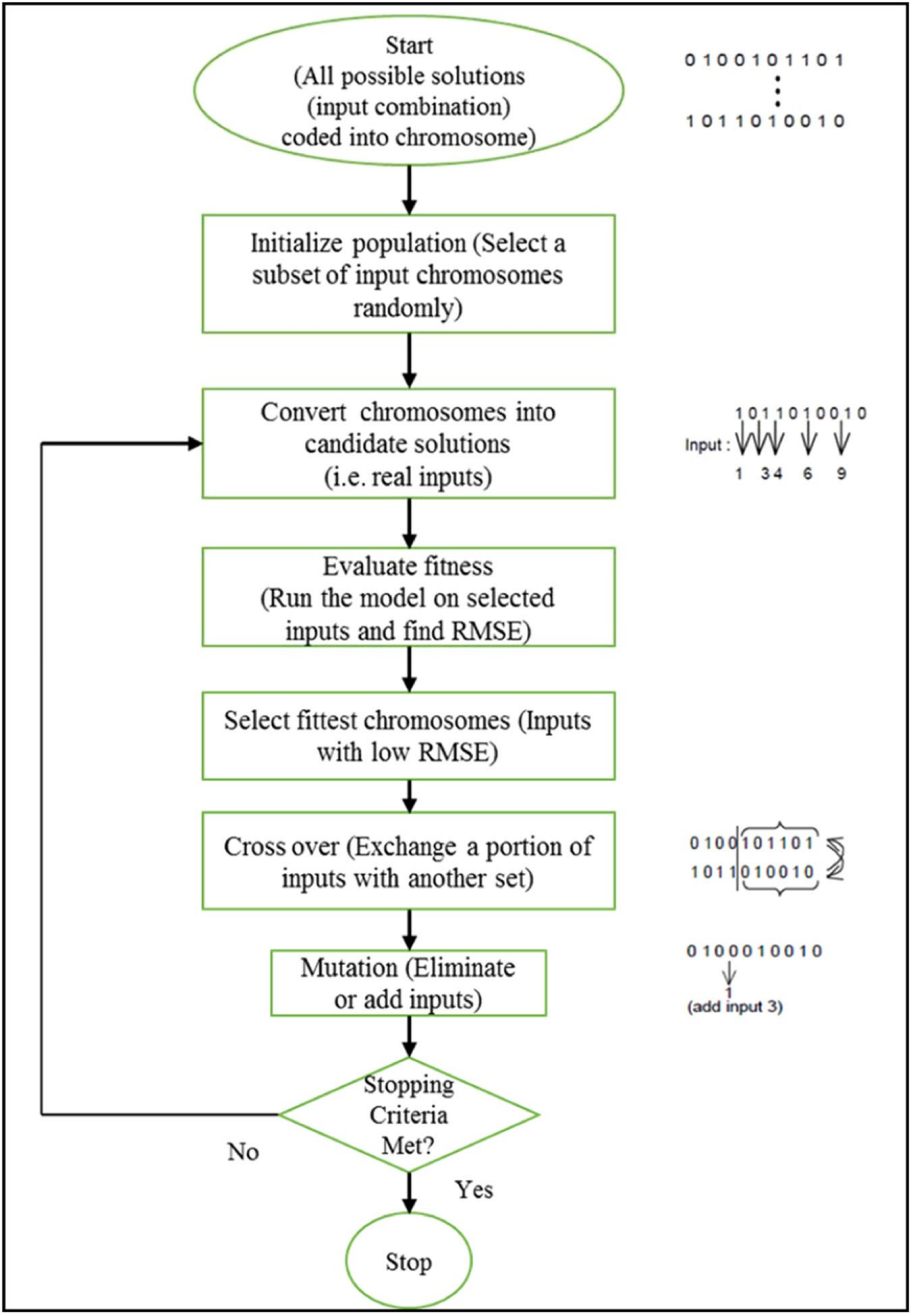
The flowchart of Genetic input selection method.

One of the robust and powerful natural evolution and selection-based optimization frameworks is the Genetic algorithm (GA)^38,39^. This framework can select the optimal input sets by searching several input variable combinations and simultaneously testing the achieved solutions. The GA searches the solution space for the best inputs that satisfy the selection criteria based on the best fitness; it is believed that the best input variables will provide the best model performance. In the GA, the individual input combinations are regarded as a possible solution. The selection of input variables using the GA-ANN model is conceptualized in Fig. 3. An optimal solution is selected based on the achieved minimum RMSE. Root Mean Square Error (RMSE) can be defined as the standard deviation of the prediction errors (residuals). Residual is a term that use to measure of how the data point far from the fit line of regression; or can defined as a measurement of how spread out these residuals are. In other words, it tells you how concentrated the data is around the best fit line. Root mean square error is generally used in climatology, forecasting, and regression analysis to verify experimental results therefore RMSE has been chosen as objective function for the genetic algorithm^40^. Three basic operations are involved in the GA searching process - selection, crossover, and mutation. Figure 4 depicts the flow chart of the GA searching operation in association with an ANN model.

The flow chart walks through the steps of input selection by highlighting each process in each step. As the first step before the selection process started, genetic encode the inputs into chromosome which is presented by brainy strings. The string is a code with length equal to the total number of input variables. The input encodes into two types if 0 refer to an absence which is unselected or 1 refer to present which is selected to involve the modeling. For example, if there are four input variables, the string {0,1,1,0} presents that inputs two and three are selected while inputs one and four are discarded.

The increment of the total number of input variables will increase the number of possible solutions. After encoding the input into strings, the GA searching process starts with an initial random set of inputs (population of chromosomes). Then evaluating the fitness of the solution by utilizing the ANN model for each input combination. The best fitness with low RMSE will be selected. The genetic select chromosome by tournament method which is one of the most common selection approaches such as Roulette Wheel and Boltzmann methods. Tournament approach applied efficiently for a wide range of problems^41^. It involves randomly picking a pair of chromosomes and then the lower RMSE will be selected to start a new generation. Since only half of the chromosomes are selected, another tournament is held using all the original chromosomes, but this time the tournament is a separate set of random chromosome pairs.

During the search process, the best and worst chromosomes are replicated in the crossover pool. A crossover occurs when two random paired chromosomes exchanges their genetic information to produce a new generation from the parent chromosomes. If the probability of a crossover is higher than a pre-set probability parameter, the information exchange between the chromosomes will progress, but if less or equal to the pre-set parameter, such an exchange will not progress. In such a situation, the two unchanged parent chromosomes will become candidate solutions in the new population. The crossover probability parameter is a key determinant for adjusting the selection pressure which is providing a convenient mechanism. This crossover probability parameter is usually pre-set at >0.5 in practice to favor fitter candidates. The final step of the genetic operation is mutation. This is the stage when the candidate solution will change their structures (0 will be replaced by 1 and vice versa). A mutation process which has been designed to give flexibility to a solution may eliminate prematurely to reconsider in the process. This might also happen to ensure the population is kept diverse in a bid to avoid premature convergence to local minima.

During mutation, the mutation probability parameter is used to decide the chances of each chromosomal bit being changed. The values of the selected bits are reversed to mutate them, ending one genetic cycle. Again, individual chromosomes are evaluated for fitness. The input for the NN model is the input selection before calculating the RMSE. The lower the RMSE of the chromosomes, the higher their fitness. This process is repeated severally until some termination criteria are reached, or until the optimal solution has been reached. For the input selection problem, the final solution is reached when the optimal set of inputs which can accurately predict the output has been established. In this study there are some internal parameters for the genetic algorithm have been setup as shown in Table 1

**Table 1:** Genetic algorithm parameters setup.

Genetic parameters	
Population size	5
Number of generations	30
Selection scheme	tournament
Tournament size	0.25
p initialize	0.5
p crossover	0.5
Crossover type	uniform

## Radial Basis Function Neural Network

Among several types of neural networks models, radial basis function neural network (RBFNN) is considered as a second popular model after FFNN model. RBFNN is a special type of feedforward network. The RBFNN architecture is consisting of three layers (input, hidden, and output). RBFNN is unlike FFNN where has a single hidden layer each processing element in this layer implements a radial basis function as activation function which is considered a nonlinear function. While each processing element in the output layer is implementing a summation function for the output of the hidden layer.

The output of the neural network is a function of inputs which is can be determined by the following equations:

1 $${\rm{Z}}({\rm{X}})={\sum }^{}w\ast \varphi (\Vert x-c\Vert )$$

where Z is the output, X is the input signals, φ is the basis function, $w$ the weight for each connection in the hidden layer, *c* the center value for the hidden neuron. Where the radial basis function $\varphi (\Vert x-c\Vert )$ is determined by the following equation:

2 $$\varphi (\Vert x-c\Vert )=exp\left\{-\frac{{\Vert x-c\Vert }^{2}}{2{\sigma }^{2}}\right\}$$

There are three important parameters in the RBFNN need to be set up which is the weight of each connection, the center of RBF, and $\sigma$ the width of the hidden unit. In the traditional RBFNN model, the centers and widths are calculated by K-mean clustering^42^. As per the Gaussian radial function, it is seen that the hidden units are more responsive to the data points nearer to the center. This sensitivity can be adjusted by using the width or the spread value. As the spread value increases, the sensitivity of the radial basis function against the input data decreases. The number of the radial basis functions in the hidden layer depend on the intricacy of the map which is modelled and not on the number of points or size of data set, which is valid while applying the multi-layer perceptron ANNs^43,44^.

## Streamflow Forecasting Model Architecture

The suggested model was applied for Nile River streamflow forecasting at the AHD utilizing historical 130-year streamflow records. The aim of this study was to forecast the streamflow of the AHD in a month using the natural data of the previous months as input combinations for modeling. Based on these considerations, a reconnaissance level analysis for the historical natural streamflow data was conducted, showing that the model could be applied using historical streamflow in the previous 11 months^45^.

From the pilot study in this work, it was observed that reliable streamflow prediction results cannot be achieved by relying on the actual streamflow from the same month of the previous years (rather than the previous months of the same year). Hence, this study relied on the nonlinear modeling capabilities of the RBNN to develop a predictive model based on the observed streamflow data for previous months of the same year. In this study, the GA was combined with an RBNN to select the optimal input pattern from the previous 12 months. Data deviation is the most important prepossessing step. In this study, the deviation 90 years training and 40 years testing have been chosen. 40 years for testing is considered a large enough as a period that contain all the pattern of streamflow over years where it is must to select target data covering most of pattern. Mathematically, the predicted streamflow ${Q}_{f}$ at month t based on the monitored streamflow ${Q}_{m}$ at the previous months (selected from the previous 12 months) can be thus presented as:

3 $${Q}_{f}(t)=f({Q}_{m}{(t-x)}_{n})$$

where ${Q}_{m}$ is the natural streamflow for the month, ${Q}_{f}$ is the forecasted inflow for the month, n is the number of the input pattern and x is the time domain (from 2 to 12); in this study, the time domain was 12. As the natural streamflow varies monthly, the current study developed individual models for each month, resulting in 12 monthly models for streamflow forecasting. The monthly natural streamflow for the 90-year period from 1871 to 1960 was utilized to calibrate the twelve models. The performance and reliability of the GA-RBNN models were examined using the monitored streamflow data from 1961 to 2000.

In this study, the choice of the number of input variables from the alternative domain is a critical step in developing the forecasting model. A. El-Shafie *et al*.^46^ conducted a comprehensive analysis of the historical streamflow data utilizing several statistical indexes including autocorrelation and cross-correlation. The study showed that the natural monthly streamflow depends on one or more records of the previous eleven natural streamflow values. In this framework, different inputs were selected utilizing the proposed GA-RBNN model during the calibration session with streamflow data from 1871 to 1960. This step is considered a pre-defined step that determines the number of historical streamflow records that will be included in the model input. Fortunately, the GA robotically selects the optimal previous month(s) (x) based on the pre-defined number of inputs (n) until reaching the performance goal (an MSE value). In this research, the parameters for the RBF-NN are chosen to be similar with those selected by A. El-Shafie *et al*.^46^.

## Evaluation Metrics

The developed predictive models were evaluated for performance using several indicators. This study investigated the proposed models for streamflow prediction efficiency using seven statistical metrics. The correlation coefficient (R^2^) is the first metric used; it is a measure of the performance pattern of a model. The relative error (RE) is the second measure; it portrays the values of the percentage error between the actual and the predicted values. The R^2^ and RE are determined using the following expressions:

4 $${{\rm{R}}}^{2}\,=\frac{{\sum }_{{\rm{t}}=1}^{{\rm{n}}}[(({\rm{Ia}})-\overline{({\rm{Ia}})})\,(\,({\rm{Ip}})-(\overline{{\rm{Ip}})})]\,}{\sqrt{{\sum }_{{\rm{t}}=1}^{{\rm{n}}}(({\rm{Ia}})-\overline{({\rm{Ia}}}){)}^{2}{\sum }_{{\rm{t}}=1}^{{\rm{n}}}{(({\rm{Ip}})-\overline{({\rm{Ip}})})}^{2}\,}}$$

5 $${\rm{RE}} \% =\left[\frac{\,({\rm{Ia}})-({\rm{Ip}})}{({\rm{Ia}})}\right]100$$

where Ia = streamflow observations, Ip = predicted output value, n = number of observations or periods the errors were predicted.

The agreement index (d), root mean square error (RMSE) and mean absolute error (MAE) are the 3^rd^, 4^th^, and 5^th^ metrics, respectively. The performance of the proposed model for the training and testing sets was determined using these statistical measures. When the values of these indicators are similar, it generally indicates good model performance. These indicators are defined thus:

6 $${\rm{d}}=1-\frac{{\sum }_{{\rm{t}}=1}^{{\rm{n}}}{(({\rm{Ia}})-({\rm{Ip}}))}^{2}}{{\sum }_{{\rm{t}}=1}^{{\rm{n}}}{(|({\rm{Ia}})-({\rm{Ip}})|+|({\rm{Ia}})-({\rm{Ip}})|)}^{2}}0\le {\rm{d}}\le 1$$

7 $${\rm{RMSE}}=\sqrt{\frac{1}{{\rm{N}}}\mathop{\sum }\limits_{{\rm{t}}=1}^{{\rm{n}}}{(({\rm{Ia}})-({\rm{Ip}}))}^{2}}$$

8 $${\rm{MAE}}=\frac{1}{{\rm{N}}}\mathop{\sum }\limits_{{\rm{t}}=1}^{{\rm{n}}}|{{\rm{I}}}_{{\rm{a}}}({\rm{t}})-{{\rm{I}}}_{{\rm{s}}}({\rm{t}})\,|$$

The mean absolute percentage error (MAPE) indicator is a measure of the prediction accuracy of the predictive model. This indicator usually expresses the accuracy as a percentage, as shown in Eq. 9. The BIAS indicator represents the mean of the individual errors and indicates whether the proposed model overestimates or underestimates the streamflow prediction and is defined by Eq. 10.

9 $$MAPE=\frac{100}{n}\mathop{\sum }\limits_{t=1}^{n}|\frac{{I}_{a}-\,{I}_{p}}{{I}_{a}}|$$

10 $${\rm{MBE}}=\frac{1}{{\rm{N}}}\mathop{\sum }\limits_{{\rm{t}}=1}^{{\rm{n}}}\left(\frac{({\rm{Ip}})-({\rm{Ia}})}{({\rm{Ia}})}\right)$$

where Ia = actual output value, Ip = predicted output value, n = number of observations or periods the errors were predicted.

## Application and Analysis

The proposed streamflow forecasting model is based on the use of past records, i.e., antecedent values, to forecast the future values from the available historical records of the time series data set. However, defining the past records that should be considered in the forecasting processes is a very significant step in such modeling to achieve the best forecasting accuracy. Input parameter selection and reduction in dimensional space are the central contributions of the current research. This could improve the modeling performance and simplify the learning processes. Eliminating the non-relative input variables from the prediction matrix can produce more reliable and robust learning procedure. Practically, this is contributing to the basic knowledge of the hydrological process where the correlated antecedent values of the historical river flow is incorporated in the learning memory of the prediction matrix. As noted above, the aim of the current research is to propose a new approach that selects the most related input variables to improve the performance learning model through eliminating redundant or irrelevant attributes that could negatively influence the model accuracy. The model structure design that integrates the RBFNN with GA to predict the monthly streamflow by using the optimal input lags.

By recalling the proposed model that was applied for each month while keeping the selection domain for each model, the input is selected from the previous 12 months. Thus, the selection is from the prior water year.

The performance of the integrated RBFNN and GA modeling in forecasting one month ahead based on the most impacted previous streamflow records during the prior water year are indicated in Tables 2 and 3 for training and testing phases, respectively. The maximum Relative Error (Max RE) was used as the main metric for determining the modeling accuracy over the testing phase. The minimum absolute error metrics (i.e., MAE, MAPE and RMSE), the best-fit-goodness coefficient of determination (R^2^) and agreement index (d) were calculated to establish minimum standards for model inter-comparison. In accordance to the reported statistical performance of the proposed predictive model over the training phase, Table 3 reveals the predictability performance for all inspected months. Based on the coefficient of determination metric, the applied predictive model demonstrated an acceptable result with R^2^ magnitudes ranged between 0.90 to 0.99, as reported by^47^. Based on the twelve-month performance results over the testing phase, more than five input variables accomplished the minimum relative error percentage, except in November, for which four input variables indicated slightly better results than those utilizing more than five input variables (August, September, October, January, and July). This shows a major advantage of the proposed model, as the high forecasting accuracy for one month might be suitable for other months in terms of the number of inputs and their relative lag-time positions with the desired month. October and July had the highest Max RE% of the modelled months, −37% and −32%, respectively. May and June had the lowest Max RE% of 7% and −9%, respectively. The relatively low level of forecasting accuracy of approximately 35%, especially for October and July, can be explained by the highly stochastic nature of the historical records for these two months. A Max RE% of approximately 10% is considered relatively high forecasting accuracy for streamflow.

**Table 2:** The evaluation metrics for training phase of the different input combinations for each month.

Month	RMSE	MAE	MAPE	MBE	d
August	2.415	1.976	11.113	0.021	0.901
September	1.789	1.435	6.581	0.006	0.948
October	1.202	0.945	6.231	−0.0006	0.968
November	0.620	0.475	5.760	0.003	0.979
December	0.241	0.177	3.071	0.0006	0.993
January	0.161	0.127	3.212	0.002	0.994
February	0.158	0.128	4.772	0.002	0.992
March	0.127	0.102	4.097	0.001	0.994
April	0.146	0.112	6.022	0.009	0.991
May	0.137	0.104	6.279	0.005	0.988
June	0.327	0.244	13.878	0.041	0.929
July	0.969	0.747	16.646	0.048	0.899

**Table 3:** The evaluation metrics for testing phase of the different input combinations for each month.

Month	RMSE	MAE	MAPE	MBE	d	Max. (RE)	R^2^
August	1.477	1.210	6.803	−0.010	0.964	−19.364	0.882
September	1.176	0.969	5.694	0.006	0.988	−23.652	0.955
October	1.001	0.864	8.804	0.037	0.977	−37.833	0.922
November	0.472	0.365	5.208	−0.003	0.982	−15.193	0.937
December	0.281	0.229	4.475	0.002	0.969	−14.950	0.886
January	0.236	0.176	3.916	−0.002	0.969	15.641	0.892
February	0.167	0.130	3.655	0.003	0.978	−11.051	0.927
March	0.152	0.118	3.669	−0.0001	0.978	11.315	0.917
April	0.184	0.149	4.107	−0.006	0.967	−11.839	0.882
May	0.125	0.101	2.853	−0.004	0.979	7.775	0.920
June	0.164	0.134	4.449	−0.001	0.976	−9.871	0.912
July	0.566	0.413	7.121	0.014	0.964	−32.878	0.876

The quantitative presentation of the minimum absolute error metrics (i.e., MAE, MAPE and RMSE) exhibited a consistent forecasting skill, with Max RE% as the main indicator considered in the analysis. Accurate forecasting was obtained for all months using five month’s attributes. Generating an accurate forecasting model for streamflow in a certain month of the year, considering the hydrological influences of the prior streamflow records, is associated with the input pattern selection and the capability of the model to detect the stochastic nature of the streamflow pattern.

The proposed model was also evaluated using other evaluation metrics, including the agreement index (d) which is a descriptive measure. Both d and R^2^ have a similar range as they vary from 0 (indicating no correlation) to 1 (indicating a perfect fit). Being that R^2^ is sensitive to the variations in the actual and predicted means and variances, it is highly sensitive to extreme values. This difficulty can be addressed by applying factor d. In addition, the agreement index was not established to be a measure of correlation. The results of the agreement index presented a remarkable harmony with the other indicators, explaining the consistency the modeling accuracy. The results in Table 3 show that the first-month lag-time is notably significant in resulting in the best performance accuracy for all months modelled regardless of the natural stochasticity of that month. The results for August prediction shows the worst performance based on of the mean absolute error and root mean square error. Such relatively poor results, particularly for this month, are due to the high stochastic river flow in the historical data.

The augmentation of RBFNN-GA is a simple explicit function for monthly streamflow forecasting based on four or more influence input variables. Table 4 shows the input combination selected by the genetic algorithm.

**Table 4:** The optimal inputs combination selected by GA for each month.

Predicted Month	Input Variables											
	January	February	March	April	May	June	July	August	September	October	November	December
January			X	X	X		X				X	X*
February	X*		X		X	X		X				
March	X	X*	X**	X				X	X	X		
April			X*		X	X	X	X	X		X	X
May	X	X	X	X*			X	X				X
June		X		X	X*		X	X				
July	X	X					X**	X	X		X	
August	X	X				X	X*		X			X
September		X	X		X	X	X	X*		X		
October		X	X	X	X				X*			X
November					X	X			X	X*		
December	X		X	X		X					X*	X**

This table includes the best inputs combination for each month to achieve the best forecasting accuracy when the number of input prior records used. The selection that provides the best forecasting accuracy of the streamflow varies for each month. One major observation from the formulas above is that one previous month is common for most months (as shown in Table 4 in X*). This observation is logical from the hydrological point of view, as the streamflow at a particular point along the river is interrelated with the streamflow of the previous month and thus the goodness of forecasting accuracy is dependent on it. The selection of one previous month is common in the selection for all months, except for July. This might be because this month (July) experience major changes in the streamflow at the AHD at the transition border of the streamflow category for low-to-average and average-to-high classes, as discussed in section (2). In addition, it can be concluded from Table 4 that only three months (March, July, and December) are affected by *t*_*−12*_ input variable (as shown in Table 4 in X**) which is represent the same month record of previous year and that might be related to the influence of the streamflow at the beginning of the water year on the streamflow at the end of the water year.

The selection of relevant inputs is a very complex process which was successfully handled by the genetic algorithm. As shown in Table 4, the selected inputs combination was variety, the selection of input revealed that every month has different input set combination than another. That is referring to the fact that GA searched for the best combination rather than best input individually or how much the input is correlated with output. With a certain combination such as January could provide a relevant information with the absence of a highly correlated values of January for previous years). Indeed, the selection of relevant inputs reduces the overfitting of the model and modelling time consumption by eliminating unnecessary input which causes a redundant in the stage of the training phase. Therefore, it can notice the variation of the selected inputs from set to another.

**Figure 5:**
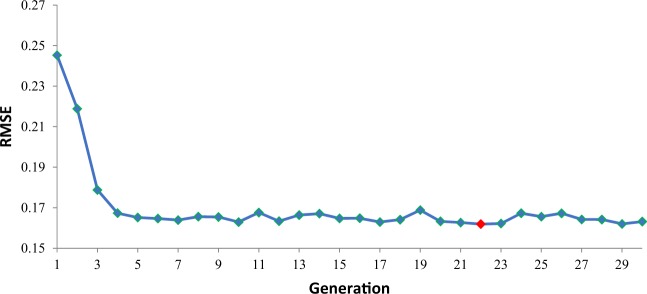
The convergence of genetic algorithm for January input selection.

The convergence of genetic algorithm for selecting the best input variables for January has been presented in Fig. 5. Where the lowest value of RMSE was 0.161 BCM within 22^nd^ generation. It is clearly seen that GA has a fast convergence within first 5 generations where the RMSE has been reduces from 0.245 BCM to 0.165 BCM.

**Figure 6:**
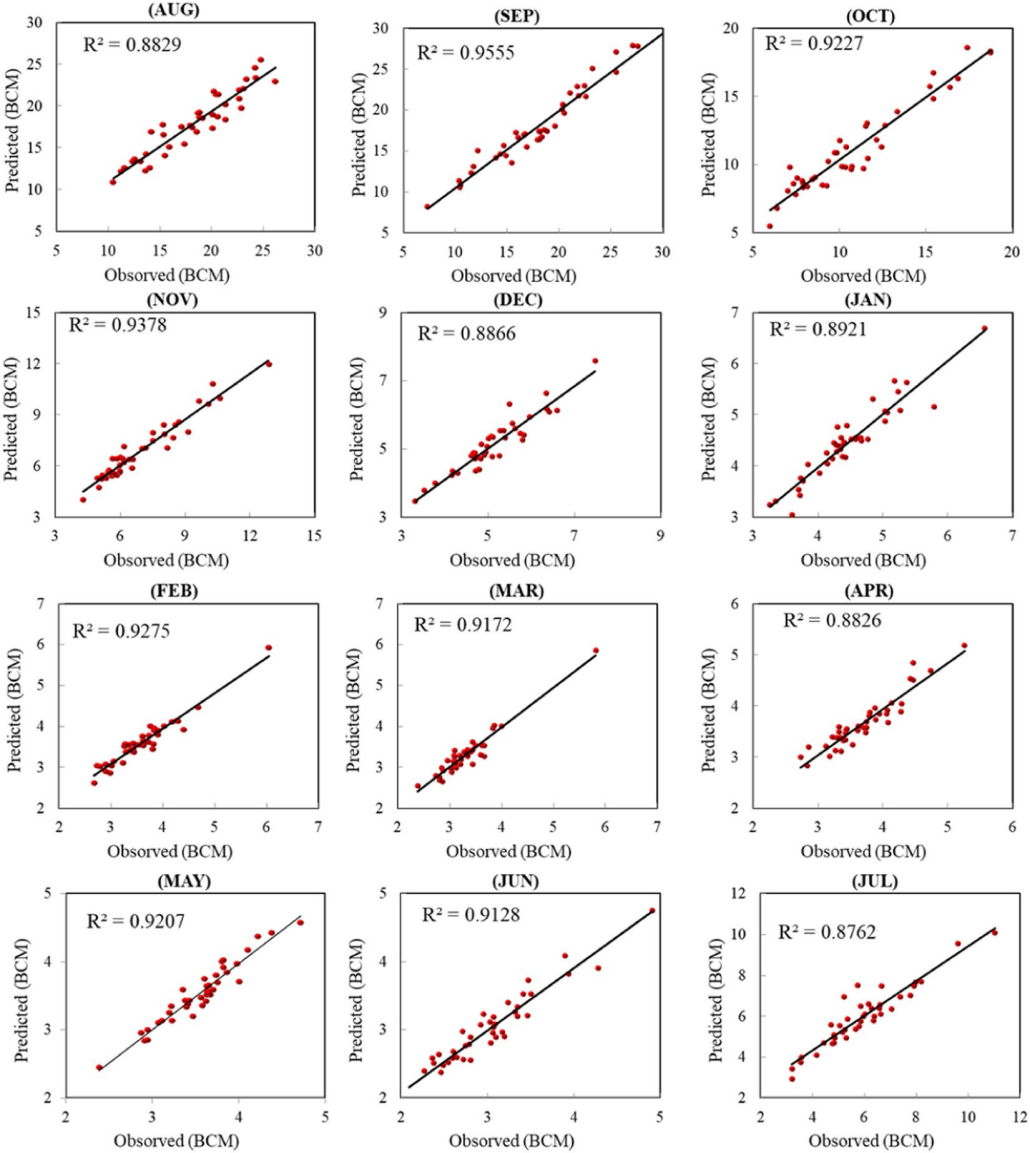
The scatter plots of the 12-months.

Furthermore, to examine the robustness of the proposed statistical input selection model with the presence of a non-stationarity streamflow pattern, Fig. 6 illustrates scatterplots for the investigated months and for each input attribute combination. Note that the scatter plot represents the correlation coefficient, which is the square of the coefficient of determination. The best results are achieved using various input variables each month, except for September and November. Within 7 inputs for September and 4 inputs for November were most suitable input combinations for predictive models and provided more accurate results for those two months. While, a relatively low correlation noticed between the actual and predicted streamflow for April and July, possibly because of a weak correlation between the inputs and the output variables for those months. Moreover, those input combinations are not sufficient for predictive models to learn the natural streamflow pattern. The improvement in the performance of the proposed model is noticeable when using more input combinations.

It is noticeable that the forecasting accuracy in some months was less than others, also there is a clear divergence from the fit line as shown in Fig. 6 where the natural flow has an inherent highly non-linear configuration. In some cases, such as August, there is a high variation of the possible streamflow pattern, which ranged from 6.5 to 29 BCM, compared to November and April, in which the streamflow pattern ranged from 4.12 to 14.4 BCM and 1 to 5 BCM, respectively. In spite of this variation of patterns, the proposed RBFNN-GA modeling could provide accurate forecasting pattern in general for streamflow that experienced high variation range of historical records that led to nonconformity of its pattern and difficulty in mimicking the pattern in modeling.

**Figure 7:**
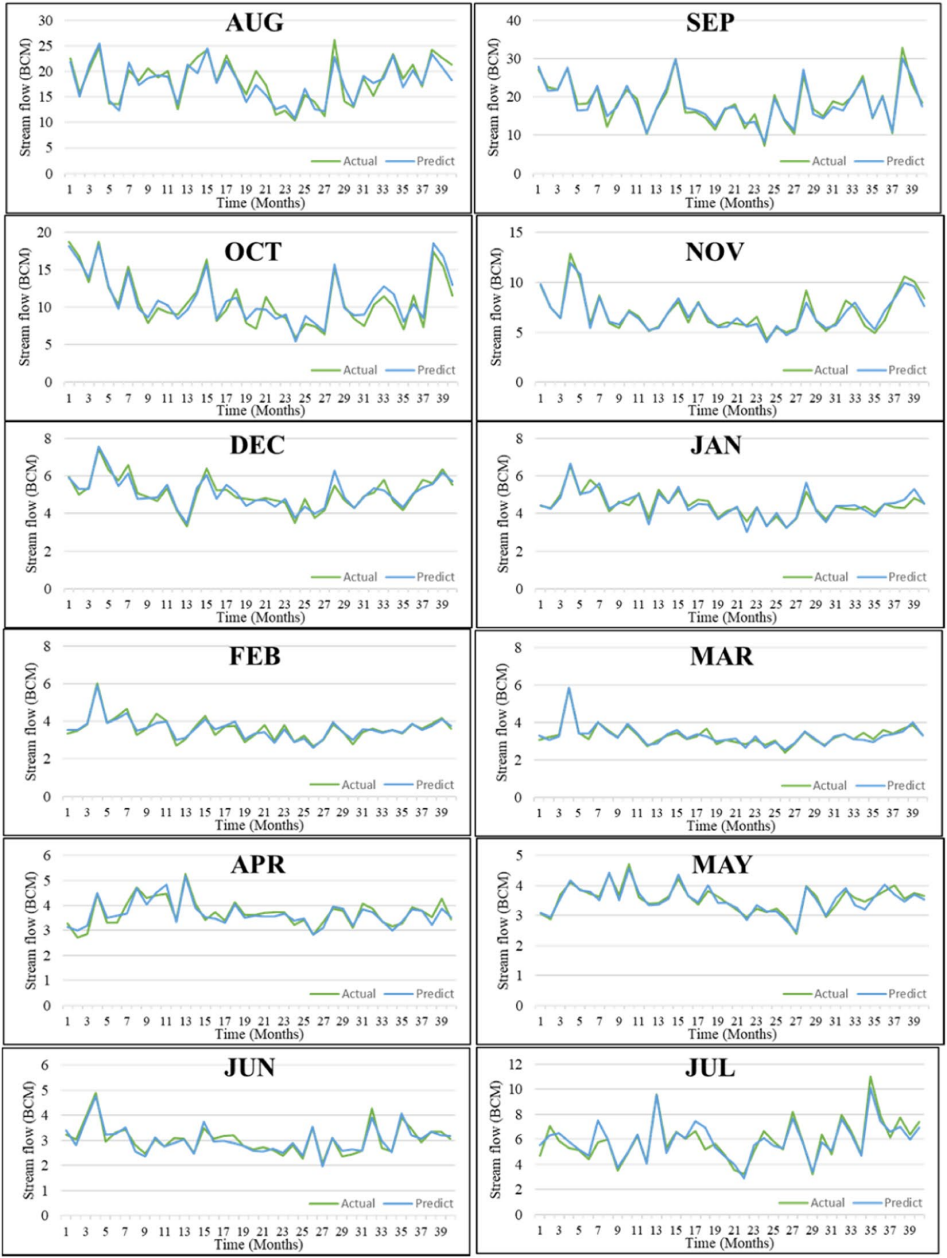
Predicted versus observed using the best input variables for each month.

Figure 7 shows the best model for each month based on the hydrograph shape, predicted and observed records to demonstrate the suggested model. There are a minor difference between the modeling results of the present study and the observed streamflow through the hydrograph shapes. The proposed method’s accuracy is analysed by comparing the predicted with the observed streamflow pattern. The RBFNN-GA method has low accuracy for July, August, and October, showing clear differences between the predict and the actual (Fig. 7). However, the predictive model provided predicted data that are relatively matched with the observed streamflow for the other months. For time series modeling and considering the hydrological context, accurate input prior lag-time selection is a critical factor to achieve accurate forecasting performance in a streamflow forecasting model. Such accurate forecasting could provide better information for water management decision-makers for better planning, river water resources system operation, and river sustainability. In modeling streamflow forecasting, the critical stage in developing such models is the selection of the proper input combinations^48^ for accurate forecasting. To model streamflow based on other hydrological parameters, sequential lag-times and auto-correlation approaches have been used. However, those approaches are very straightforward in allocating the input model parameters based on the regression function; in addition, there is no elimination of irrelevant input combinations.

The proposed approach was successfully developed to perform input pattern selection. However, the proposed RBFNN-GA is still incapable of providing a high level of accuracy for streamflow forecasting because the predictor component of the model (RBFNN) is vulnerable to identifying the stochastic constituent in the data. The proposed model was unsuccessful in providing a high level of accuracy for two months. In this context, such a drawback could be overcome by integrating the GA with other modeling methods that have been successful in detecting highly stochastic components of data with a high range of variation.

In conclusion, the integrated RBFNN with GA presented alternative input determination method to the state-of-the-art methods such as (i) cross-correlation analysis of potential predictors, which is based on linear analytical assumptions and may be inappropriate for complex, non-linear hydrological systems; and (ii) the conventional trial and error procedure. In addition, GA could be integrated with other advanced modeling methods.

To ensure the validity of the proposed model, the proposed model (RBFNN-GA) was compared with a previous study that addressed streamflow forecasting in the Nile River. First, an analysis was conducted between the RBFNN-GA and the RBF-NN introduced by El-Shafie *et al*. (2009). The comparison was focused on the best results provided by the current and previous models. Two different statistical indicators were selected to examine the models in the testing stage, including the root mean square error (RMSE) and mean absolute error (MAE). These indicators were selected to present the reduction of the error between the predicted and actual streamflow.

Table 5 presents these statistical criteria for both models for every month and the accuracy improvement (AI%) indicator. The accuracy improvement was measured for both indicators to show the differences between the proposed RBFNN-GA model and the RBF-NN model; the accuracy improvement could be expressed as follows:

$$AI \% =\frac{{I}_{previous}-\,{I}_{current}}{{I}_{current}}$$

where ${I}_{current}$ is the statistical indicator given by the current model (RBFNN-GA) and ${I}_{prvious}$ represents the same statistical index given by previous models (RBF-NN). Negative values of AI% indicate an enhancement of the current model compared to RBF-NN.

**Table 5:** Comparison between the RBFNN-GA and previous study according to the MAE and RMSE indicators values.

Month	RBFNN-GA		RBF-NN by^46^		Accuracy Improvement (AI%)	Accuracy Improvement (AI%)
	MAE	RMSE	MAE	RMSE	MAE	RMSE
August	1.210	1.477	2.4	0.552	98.34	−62.62
September	0.969	1.176	0.85	0.582	−12.28	−50.51
October	0.864	1.001	1.82	2.03	110.64	102.79
November	0.365	0.472	0.92	1.71	152.05	262.28
December	0.229	0.281	0.32	0.67	39.73	138.43
January	0.176	0.236	0.53	0.54	201.13	128.81
February	0.130	0.167	0.28	0.37	115.38	121.55
March	0.118	0.152	0.22	0.42	86.44	176.31
April	0.149	0.184	0.18	0.28	20.80	52.17
May	0.101	0.125	0.21	0.51	107.92	308
June	0.134	0.164	0.57	1.14	325.37	595.12
July	0.413	0.566	3.25	1.02	686.92	80.21

Table 5 shows that the RBFNN-GA algorithm provided highly accurate statistical indexes for every month compared to RBF-NN, except for August and September. The modelling results show some failure for two months which is August and September, those two months characterized by high streamflow comparing with other months over the years, thus it can be concluded that the proposed model has low accuracy for predicting the high streamflow of August and September. However, the accuracy improvement using the current model (i.e., RBFNN-GA) was highly significant compared to the previous model for other months.

## Conclusion

In this study, a methodology based on an integrated radial basis neural network model and a genetic algorithm was investigated for optimal determination of the lag time of highly non-linear long-term streamflow forecasting. The aim of the applied method was to overcome the drawbacks of classic data-driven input determination. Instead of a trial and error procedure or linear auto-correlation function methods, we presented a method of input parameter selection for a machine learning model. The proposed model structure comprises the radial basis neural network model coupled with the Genetic algorithm (RBFNN-GA) and was applied to natural streamflow to develop one-month-ahead streamflow forecasting. The model provided reliable results and achieved an acceptable level of accuracy in forecasting natural streamflow. The achieved accuracy is promising to consider this model in applications in weather forecasting or prediction of other hydrological parameters. The current state of the art in streamflow forecasting for accuracy enhancement includes a predictive model (i.e., RBFNN) and optimized search (i.e., GA), which have had limited success in forecasting long-term river streamflow. Thus, there is still a need for river streamflow accuracy enhancement techniques that can mimic streamflow non-stationary patterns for short- and long-term errors. In addition, the suitability of the RBFNN-GA algorithm to accommodate different climate parameters should be evaluated, as it might be more advantageous for river case studies to achieve better forecasting accuracy. Increasing the input variables for modeling may allow for the optimized model to select more accurate input combinations. The advantage of the GA algorithm is the ease of integration with other predictive hydrological models for river streamflow. Utilizing climate parameters (rainfall, temperature, and other parameters) in the input combinations may improve the modeling accuracy. Feeding the AI methods the optimal input combinations using GA might result in a model that can detect streamflow patterns and attain an acceptable level of accuracy.
